# Reduced-representation sequencing identifies small effective population sizes of *Anopheles gambiae* in the north-western Lake Victoria basin, Uganda

**DOI:** 10.1186/s12936-018-2432-0

**Published:** 2018-08-06

**Authors:** Rachel M. Wiltshire, Christina M. Bergey, Jonathan K. Kayondo, Josephine Birungi, Louis G. Mukwaya, Scott J. Emrich, Nora J. Besansky, Frank H. Collins

**Affiliations:** 1 Eck Institute for Global Health, Department of Biological Sciences, University of Notre Dame, Notre Dame, IN 46556 USA; 2 Departments of Anthropology and Biology, The Pennsylvania State University, University Park, PA 16802 USA; 3 Division of Entomology and Vector Biology, Uganda Virus Research Institute, Plot No. 51-59, Nakiwogo Road, Entebbe 49, Uganda; 4 Department of Electrical Engineering and Computer Science, University of Tennessee, Knoxville, TN 37996 USA

**Keywords:** *Anopheles gambiae*, Single nucleotide polymorphism, SNP, RADseq, Population structure, Effective population size, Ssese Islands, Uganda

## Abstract

**Background:**

Malaria is the leading cause of global paediatric mortality in children below 5 years of age. The number of fatalities has reduced significantly due to an expansion of control interventions but the development of new technologies remains necessary in order to achieve elimination. Recent attention has been focused on the release of genetically modified (GM) mosquitoes into natural vector populations as a mechanism of interrupting parasite transmission but despite successful in vivo laboratory studies, a detailed population genetic assessment, which must first precede any proposed field trial, has yet to be undertaken systematically. Here, the genetic structure of *Anopheles gambiae* populations in north-western Lake Victoria is explored to assess their suitability as candidates for a pilot field study release of GM mosquitoes.

**Methods:**

478 *Anopheles gambiae* mosquitoes were collected from six locations and a subset (N = 96) was selected for restriction site-associated DNA sequencing (RADseq). The resulting single nucleotide polymorphism (SNP) marker set was analysed for effective size (N_e_), connectivity and population structure (PCA, F_ST_).

**Results:**

5175 high-quality genome-wide SNPs were identified. A principal components analysis (PCA) of the collinear genomic regions illustrated that individuals clustered in concordance with geographic origin with some overlap between sites. Genetic differentiation between populations was varied with inter-island comparisons having the highest values (median F_ST_ 0.0480–0.0846). N_e_ estimates were generally small (124.2–1920.3).

**Conclusions:**

A reduced-representation SNP marker set for genome-wide *An. gambiae* genetic analysis in the north-western Lake Victoria basin is reported. Island populations demonstrated low to moderate genetic differentiation and greater structure suggesting some limitation to migration. Smaller estimates of N_e_ indicate that an introduced effector transgene will be more susceptible to genetic drift but to ensure that it is driven to fixation a robust gene drive mechanism will likely be needed. These findings, together with their favourable location and suitability for frequent monitoring, indicate that the Ssese Islands contain several candidate field locations, which merit further evaluation as potential GM mosquito pilot release sites.

**Electronic supplementary material:**

The online version of this article (10.1186/s12936-018-2432-0) contains supplementary material, which is available to authorized users.

## Background

The World Health Organization (WHO) estimated that 216 million cases of malaria occurred globally in 2016 resulting in approximately 445,000 deaths [1]. Although existing malaria control interventions have reduced mortality figures significantly in the last decade, the development of innovative mosquito vector [2] and *Plasmodium* parasite control technologies [3, 4] will be required to reduce incidence rates below the threshold that sustains transmission if the ultimate goal is malaria elimination, a target difficult or impossible to achieve by traditional control tools in regions with intense malaria transmission [2, 5].

Although challenging, and technically complex to construct, genetically modified (GM) mosquitoes as an alternative vector control tool have increasingly gained attention over recent years in parallel with the genome sequencing advancement of the major *Anopheles* vectors [6–8].

The principal goal of creating GM *Anopheles* mosquitoes is to decrease their vectorial capacity to transmit *Plasmodium* parasites, either through population suppression or replacement [9], by rendering them refractory to infection [10, 11] and examples of successful genetic constructs and drive systems have been demonstrated [12–14]. If transgenic *Anopheles* vector populations are to be established as part of a malaria control intervention then these achievements must be successfully translated from bench to field. The first step in realising this strategy is to obtain a detailed understanding of the genetic structure of the natural populations into which the transgenic construct and gene drive system will be introduced as identifying levels of gene flow (genetic exchange) and the effective population size (N_e_) will be critical to predicting the dispersal and maintenance of a transgene.

In sub-Saharan Africa, *Anopheles gambiae* is an important mosquito vector of the *Plasmodium* malaria parasite species, which infect humans. Its population structure across the African continent has been extensively studied and was unexpectedly shallow [15–18]. Comparison of allozymes (mean F_ST_ 0.036) and microsatellites (mean F_ST_ 0.016) revealed extensive inter-population gene flow over a 6000 km distance [15] that contrasted sharply with those across the Kenyan Rift Valley Complex (KRVC) (mean microsatellite F_ST_ 0.104: mean mitochondrial DNA F_ST_ 0.176), a much shorter distance of 700 km, which was attributed to the KRVC acting as a physical barrier to gene flow [17, 18]. Oceanic island studies of *An. gambiae* population structure have also demonstrated varying degrees of differentiation that range from considerable genetic exchange in the Bijagós archipelago of Guinea-Bissau (F_ST_ 0–0.019) to restricted gene flow between the Comoros Islands (F_ST_ 0.093–0.126) [19]. Despite the desirable genetic characteristics observed in *An. gambiae* populations of the Comoros, they are not well suited to the frequent monitoring that transgenic field studies require being nearly 1000 km offshore. A comparably appropriate alternative would be a lacustrine setting with multiple islands in a malarious region: Lake Victoria.

There have been two previous *An. gambiae* population genetic studies in Lake Victoria. Chen et al. [20] developed six microsatellites from five island and six mainland populations in Western Kenya and showed that there was a low but statistically significant genetic structure (mean F_ST_ 0.0010–0.019, p < 0.001), which also supported a significant correlation between geographic distance and genetic differentiation (Mantel: p < 0.001). Kayondo et al. [21] examined genetic structure in *An. gambiae* populations in the Ssese Islands, the focus of the present study, using microsatellite markers with temporal sampling that also demonstrated low but statistically significant genetic differentiation (mean F_ST_ 0.014–0.105, p < 0.05). In contrast with Chen et al. [20]; however, that study found no support for the isolation-by-distance model (Mantel: p = 0.787) and concluded that the Ssese populations varied as a result of: (i) restricted gene flow (due to separation from the mainland by water); (ii) small N_e_, and (iii) temporal instability, which, combined, had provided these mosquitoes with the opportunity to differentiate genetically.

This study aimed to follow up that of Kayondo et al. [21] by determining the current genetic structure in the same *An. gambiae* populations using recent advances in next-generation sequencing technologies. Single nucleotide polymorphism (SNP) markers were selected to capture high-density sequence variation due to their: abundance in the *An. gambiae* genome [22], lower mutation and genotyping error rates, adaptability to high-throughput assays, and utility in creating an informative marker panel applicable to future discovery and research enquiries. Restriction site-associated DNA sequencing (RADseq) [23, 24] was applied as the most economical high-resolution approach to generate a genome-wide SNP marker set for this important malaria vector in the Lake Victoria region.

## Methods

### Study area

**Fig. 1:**
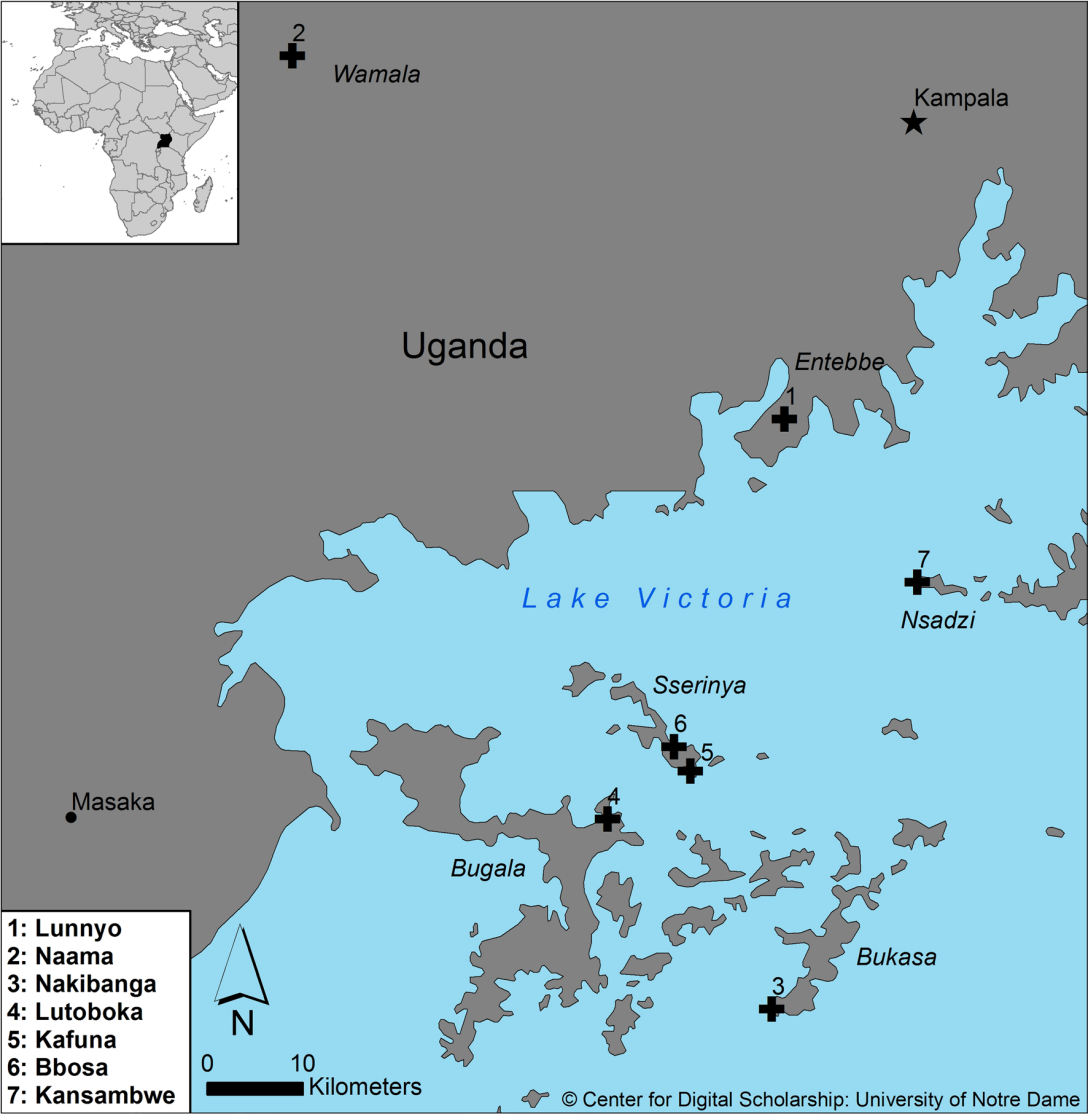
Study site locations in north-western Lake Victoria and southern Uganda peninsular. Inset top left: Uganda is highlighted in black to illustrate its location within continental Africa. Inset bottom left: Key lists entomological sampling site locations corresponding to numbered black crosses in the main picture. The black star marks Kampala, the capital city

The study area is located in the Ssese Islands, an archipelago in the north-western division of Lake Victoria, southern Uganda (Fig. 1). Each of the 84 islands varies in size i.e. the largest, Bugala, is 105 km^2^ while some are merely islets of rock, creating a total land coverage area of 454.8 km^2^. The islands share a general topographical characteristic in that they rise as gentle slopes from lake level (1220 m above sea level (ASL)) to central flat-topped ridges at a maximum elevation of 1260 m ASL (Kalangala Town, Bugala) [25, 26]. The climate is equatorial. There are two wet seasons: a main one from March–May, and a lesser one in November–December, but rainfall occurs monthly (mean 140 mm), which is reflected by the highest recorded annual precipitation rates (2000 mm+) in Uganda [25–27]. Annual temperatures range from 18.3 °C (February) to 27.2 °C (August) with relative humidity being lowest in February (68%) and highest in November (> 94%) during the warmer rainy season.

The Ssese Islands fall under the administrative jurisdiction of the Kalangala District (Kalangala) local government. The most recent census [28] lists the population as 54,293—a 56% increase from the previous official figure of 34,800 (2002), which is most likely attributable to the palm oil production and tourism industries that have recently been established on Bugala. Populations tend to be clustered in small communities along the shoreline since fishing is the major economic activity. Kalangala has some of the highest malaria incidence rates in Uganda with 208 cases per 1000 population having laboratory confirmed, and/or clinically diagnosed malaria infections [29]. In the most vulnerable group—infants under the age of 5 years—annual prevalence (44%) also indicates one of the highest national transmission rates [30], which is most likely a reflection on the lack of vector control activities in the region. There has never been an organized IRS campaign as part of a government-supported malaria control effort, and the first distribution of LLINs (to pregnant women, and children less than 5 years of age) did not take place until 2009/2010 [31]. A dedicated National Universal Coverage campaign has since distributed nearly 50 million LLINs nationwide with Kalangala receiving their allocation in November 2017 [31]. Prior to the mass net distributions, 61% of households in Kalangala were recorded as owning at least one LLIN but usage by all groups was approximately half (household population: 44%, pregnant women: 56%, and children less than 5 years of age: 50%) [30].

*Anopheles gambiae* sensu lato (s.l.) mosquitoes were sampled from seven sites: (1) Kansambwe, Nsadzi (NZ); (2) Lutoboka, Bugala (BL); (3) Kafuna, Sserinya (SYK); (4) Bbosa, Sserinya (SYB), and (5) Nakibanga, Bukasa (BK) from the islands, and (6) Lunnyo, Entebbe (EB), and (7) Naama, Wamala (WL) from the mainland, reflecting the microsatellite populations analysed by Kayondo et al. [21]. The villages in the Ssese Islands are inhabited by human populations that vary in size from hundreds (i.e. Kafuna, Bbosa) to thousands (i.e. Kansambwe, Lutoboka) of individuals. In addition to the continuous fishing traffic that is typically seen at the boat-landing sites, there is notable marine transportation between the mainland and Lutoboka (BL) via an official ferry route and, also the smaller water-taxi type services that frequently traverse the lake i.e. Entebbe–Kansambwe (NZ). Entebbe sits on a southern peninsular extending into Lake Victoria. It differs from the other sampling sites in that it is highly populated (2014 census: 69,430) [28] and urbanised. Naama, located by the shores of the inland Lake Wamala (64 km north-west of Entebbe), is an agricultural village of similar size to Kafuna and Bbosa (Sserinya). The geographic distances between all of the sampling sites are; however, outside of the known flight range of *An. gambiae* s.l. [32], meaning that migration between populations under the mosquitoes’ own power, whilst possible if wind-assisted, is unlikely. Details of longitude, latitude and geographic distances between entomological sampling sites are listed in Additional file 1.

### Entomological sampling

Collections were made at random intervals between July and October 2012. Indoor-resting (IR) adult females were collected from houses or common buildings within a 3 km radius of the boat-landing site for each island and the Entebbe locations. Sampling at Wamala was conducted with the same criteria but used Naama village as a centre point. Buildings were constructed from a combination of mud or wooden walls and thatched or corrugated sheet metal roofs. IR adult females were captured between 06:00 and 10:00 a.m. via battery-powered mechanical aspirators. If insufficient IR adult females were collected then aquatic larval samples were sourced from 5 to 10 surrounding breeding sites (type varied by location but generally small pools, puddles or abandoned boats), taken back to the laboratory and reared into adults in the water that they were collected in. This water was supplemented with mice feed pellets as required.

### Species identification and preservation

*Anopheles gambiae* s.l. mosquitoes were morphologically identified from other anopheline species based on the identification keys of Gillies and de Meillon [33]. Female specimens were individually preserved in 80% ethanol prior to transportation to the University of Notre Dame (USA). Molecular identification of *An. gambiae* and *Anopheles arabiensis*—the other important malaria vector in the *An. gambiae* s.l. complex—was determined by Scott et al. [34] using legs and/or wings. Only specimens identified as *An. gambiae* were processed further.

### Genomic DNA extraction

Genomic DNA was extracted from individual mosquitoes using a laboratory stock solution of 2% cetyltrimethyl ammonium bromide (CTAB). Each specimen was placed in an Eppendorf tube containing 200 μl of CTAB and electrically homogenized with a sterile conical Teflon pestle. RNA was removed from the homogenate by adding 20 μl RNAse A (10 mg/ml) (laboratory stock) and leaving it to incubate at room temperature (RT) for 5 min. Proteins were removed with the addition of 20 μl of Proteinase K (20 mg/ml) (Qiagen GmbH, Germany). The solution was briefly vortexed (1–2 s) on a low setting (3–4) to encourage maximum digestion and incubated at 56 °C for 1 h. Exoskeleton and other cellular detritus were pelleted by RT centrifugation at 14,000 rpm for 5 min. The supernatant was transferred to a Phase Lock Gel tube (5 Prime GmbH, Germany) with 250 μl of UltraPure™ Phenol:Chloroform:Isoamyl alcohol (25:24:1, v/v) (Invitrogen Corporation, Canada) for extraction via the standard Phenol:Choloroform method [35]. Samples were quantified with the QuantiFluor dsDNA System (Promega Corporation, USA) to ensure accuracy. 16 samples of the highest concentration from each of the six locations were selected for analysis.

### RAD library construction and sequencing

RADseq libraries were prepared as per Parchman et al. [36], which was modified to incorporate paired-end (PE) chemistry. All samples were digested with *Eco*RI and *Mse*I restriction enzymes (NEB, Inc.) and incubated at 37 °C for 2 h, then 65 °C for 20 min with the heated thermal cycler lid (Eppendorf AG) at 105 °C followed by a 4 °C hold without the lid. The digested DNA fragments were then ligated to the *Eco*RI and *Mse*I adapters with T4 DNA Ligase (NEB, Inc.).

The *Eco*RI adapter sequences consisted of Illumina adapters and primer sequences, a unique 8–10 nucleotide (nt) barcode created by a Python script [37] that permits identification of the origin of each sequencing read, a protector base to prevent further restriction site cutting, and additional bases to match the sticky ends of the cut sites: (*Eco*RI adapter sequences: 5′-CTCTTTCCCTACACGACGCTCTTCCGATCT + 8–10 nt barcode + C-3′ and 3′-TGTGAGAAAGGGATGTGCTGCGAGAAGGCTAGA + 8–10 nt barcode + G-5′). The *Mse*I adapter sequences were modified from the original protocol to facilitate PE sequencing strategy and also consisted of Illumina adapters and primer sequences, a protector base, and additional sticky end-matching bases: (JT-*Mse*I1: 5′-GCAGAAGACGGCATACGAGATCGTGATGTGACTGGAGTTCAGACGTGTGCTCTTCCGATC-3′ and JT-*Mse*I2: 5′-TAGATCGGAAGAGCACACGTCTGAACTCCAGTCACATCACGATCTCGTATGCCGTCTTCTGCTTG-3′). The DNA plate was then incubated in a thermal cycler at 16 °C for 2 h with the heated lid at 20 °C followed by a 4 °C hold without the lid.

Adapter-ligated fragments were amplified using Illumina PCR primers, which were designed to amplify only those DNA sequences with the *Eco*RI- and *Mse*I-ligated adapters. Modifying the *Mse*I adapter to facilitate PE sequencing necessitated modification of the reverse Illumina PCR primer, accordingly: (Illpcr1: 5′-AATGATACGGCGACCACCGAGATCTACACTCTTTCCCTACACGACGCTCTTCCGATCT-3′; JT-Illpcr2: 5′-CAAGCAGAAGACGGCATACGAGATCGTGATGTGACTG-3′). This step was performed running two separate 20 μl PCR amplification reactions for each adapter-ligated DNA sequence to ameliorate stochastic differences in the resulting reaction products. Both PCR plates were incubated in a thermal cycler using the following profile: 98 °C for 30 s; 30 cycles of 98 °C for 20 s; 60 °C for 30 s; 72 °C for 40 s, and a final extension at 72 °C for 10 min with the heated lid at 105 °C followed by a 4 °C hold without the heated lid.

Reaction products were pooled and purified with Agencourt AMPure XP (Beckman Coulter, Inc.) magnetic beads and size-selected using the automated BluePippin (Sage Science, Inc.) system, which recovered eluted DNA fractions between 400 and 500 base pairs (bp). Sequencing was accomplished in a single lane run on an Illumina HiSeq 2000 (v.1.5) machine at the University of California-Davis, Sacramento, USA via the Beijing Genomics Institute.

### Determination of 2L chromosomal karyotype

Molecular karyotyping of the 2La inversion was conducted as per White et al. [38] with a modified thermal cycler profile as follows: 94 °C for 2 min; 30 cycles of 94 °C for 30 s; 58 °C for 30 s; 72 °C for 45 s; a final extension at 72 °C for 5 min, and a 4 °C hold. The resulting products were analysed on 1.5% agarose gels stained with SYBR Safe (Life Technologies Corp.).

### Bioinformatics processing

After quality checking of the sequence data in FastQC v.0.10.1 [39], Illumina sequencing adapters were removed using Trimmomatic v.0.30 [40]. RAD barcodes were stripped from the reads and replaced by unique identifiers specific to each individual mosquito by a custom Python script, Trimmer [41]. Sequence reads were then aligned against the AgamP4 reference genome [42] using Burrows-Wheeler Alignment (BWA) v.0.6.2 [43] prior to variant (SNP) calling and annotation with UnifiedGenotyper in GenomeAnalysisToolKit (GATK) v.3.3.0 [44]. High quality SNP calls used in downstream analysis were obtained firstly through the application of the hard-filtering parameters as described in Alternate Protocol 2 of the GATK best practices pipeline [44] followed by a stricter filtering of the dataset as per Fontaine et al. [45] using VCFtools v.0.1.15 [46]. A detailed description of the pipeline, including parameters, can be found in Additional file 2.

Individuals were pruned from the dataset on the basis of kinship and/or missing data. Familial relationships were assessed by pairwise comparison of kinship coefficients estimated using the (--relatedness) [47], and (--relatedness2) [48] parameters in VCFtools v.0.1.15 [46]. Relationships that identified individuals as full siblings resulted in their removal from the dataset. Missing genotypes were assessed on an individual basis using the (--missing-indv) parameter in VCFtools v.0.1.15 [46]. Any found to have > 80% missing data were also discarded.

### Population genetics

Population structure was visualized by two methods: (1) principal component analysis (PCA) using the software packages PLINK v.1.9 [49] and R v.3.2.1 [50], and (2) ancestry fractions computed from maximum-likelihood estimates using population allele frequencies and genotype probabilities as parameters of a statistical model in the program ADMIXTURE v.1.23 [51]. The number of ancestral populations (*K*) with which to run the model was chosen by a cross validation (CV) procedure that identified the lowest error value for which the model had the best predictive accuracy.

Genetic differentiation between populations was quantified by Wright’s fixation indices (pairwise F_ST_) [52] using Weir–Cockerham weighted multiallelic estimates [53] in VCFtools v.0.1.15 [46]. Individuals with > 80% missing data were removed to ensure accuracy since simulations have shown that restricting loci to those with complete genotypes results in a near true F_ST_ distribution [54]. To test whether variation was attributable to isolation-by-distance [55], a linear regression model of pairwise population differentiations (F_ST_/(1 − F_ST_)) against logarithmic transformed geographical distances [56] was created in R v.3.2.1 [50] using a generalized linear model (GLM) function. Statistical significance between the spatial and genetic sets of distances was measured by the Mantel test with 9999 permutations [57]. Estimates of contemporary N_e_ were obtained using the linkage disequilibrium (LD)-based method LDNe [58] of NeEstimator v2.01 [59] with a minor allele frequency screen of 5%.

## Results

### Species identification and dataset composition

479 individuals were molecularly identified as *An. gambiae* and one as *An. arabiensis* (from the Entebbe collection site). A total of 373,099,980 reads were generated by the Illumina HiSeq 2000 platform. After demultiplexing the raw data of sequencing adapters, barcodes, *Eco*RI and *Mse*I restriction cut sites and protector bases, a total of 172 million reads averaging 1.6 million per mosquito (N = 96) were retained for genomic alignment. 103 million forward reads (86.2%), approximately 83 bases in length, successfully mapped to the AgamP4 reference genome [42], which were then used in downstream analyses.

Examination of kinship identified a large number of familial relationships between individuals in the Sserinya (SY) and Bugala (BL) populations. Eleven individuals from SY and three individuals from BL were excluded from further analysis on the basis that their full and half kinship could confound the data at each site and in comparison with others. Three individuals from the Bukasa (BK) population with > 80% missing genotype information were also removed from the dataset (N = 79). Kinship coefficient estimates and percentage missing data values per individual are listed in Additional file 3.

### Chromosomal mapping and distribution of SNPs

After high quality SNP calling, application of hard filters to, and pruning from, the dataset, a total of 5175 SNPs were identified and mapped to the AgamP4 chromosomes [42] as follows: X (n = 347), 2L (n = 1078), 2R (n = 1514), 3L (n = 936), 3R (n = 1204), and mitochondrial (n = 1). 95 SNPs were unable to be assigned to any chromosome (UNKN) but were included in a population genetic analysis when the collinear genome was being explored. The UNKN SNPs are most likely physically located in the highly repetitive pericentromeric regions [60], which are challenging genomic positions to assemble and map.

### Population structure

**Fig. 2:**
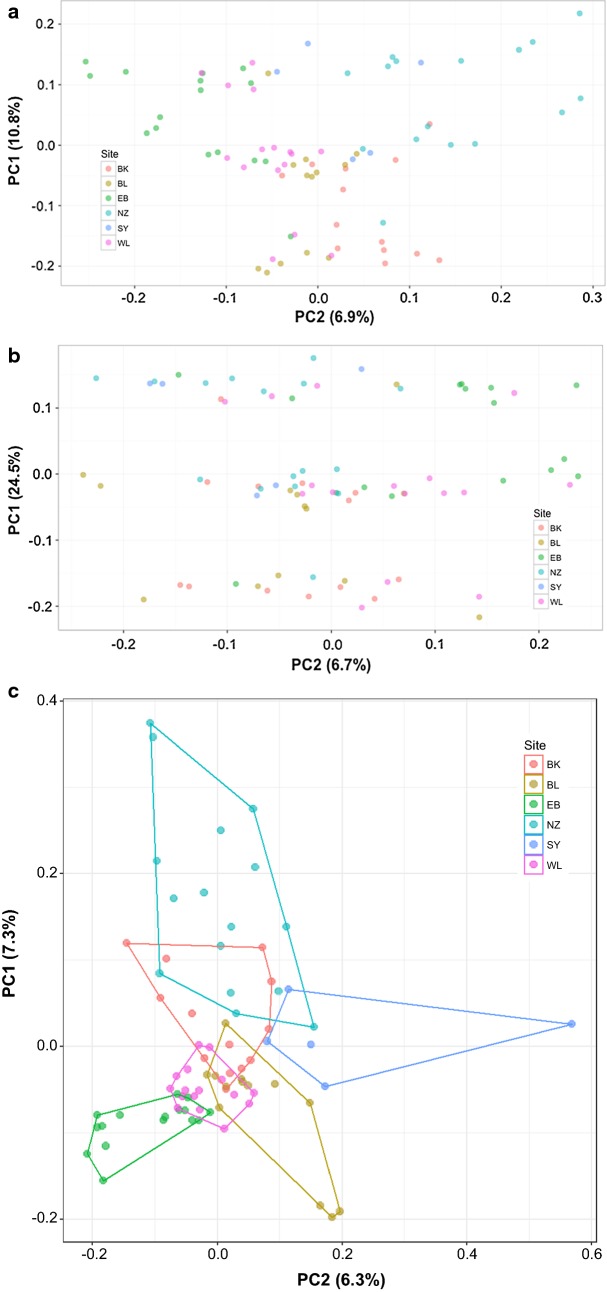
Principal component analysis (PCA) plots of the *Anopheles gambiae* genome-wide SNP dataset (N = 79). In **a**–**c** each dot represents an individual mosquito that was sequenced by RADseq technology. Entomological sampling sites are color-coded according to the legend as follows: BK (Bukasa); BL (Bugala); EB (Entebbe); NZ (Nsadzi); SY (Sserinya), and WL (Wamala). The first principal component (PC1) and its percentage variance are represented by the *y*-axis and, likewise, the *x*-axis represents the second principal component (PC2) and its percentage variance. **a** Plot of 5175 SNPs mapped to the AgamP4 genome [42]. **b** Plot of 1078 SNPs mapped to chromosome 2L [42]. **c** Plot of 4097 SNPs mapped to the AgamP4 genome [42] after removal of chromosome 2L SNPs

Visualization of population structure by PCA illustrated how the SNPs genetically clustered within and between collection sites. Genome-wide analysis (n = 5175) showed individuals clustering into three discrete groups on the first principal component (PC1) in a non-geographical configuration (Fig. 2a), which was also observed in the chromosome 2L (n = 1078) PCA (Fig. 2b), a pattern likely driven by polymorphism with respect to the 2La inversion (Additional file 4) [61]. When 2L SNPs were removed from the data set, or when other chromosome arms were analysed individually, genetic structure showed individuals generally clustering in concordance with their geographic origin (Fig. 2c; Additional file 5). Since the 2La inversion is known to confound population genetic structure [8, 61], chromosome 2L SNPs were removed from the dataset (n = 4097).

Population structure was additionally tested by model-based estimation of ancestry using the software program ADMIXTURE v.1.23 [51]. CV testing for each chromosome produced error estimates that indicated the populations shared only one ancestry except for 2L where *K *= 2 was the most likely number of fractions (Additional file 6). This was in line with the expectation that populations would cluster into their chromosomal inversion arrangements (2L+ ^a^/+ ^a^, 2La/a and 2L+ ^a^/a) rather than geographic locations for this region of the genome (Additional file 4).

### Genetic differentiation

Pairwise F_ST_ comparisons between the populations were computed for all mapped SNPs according to Weir and Cockerham [53] weighted estimates. Median values were used in the analysis since a null distribution histogram showed that they were not normally distributed. Moderate amounts of genetic differentiation were observed between most of the populations (median F_ST_ 0.0342–0.0903) for the 4097 SNPs across the collinear genome after removal of the SNPs on chromosome 2L (Table 1).

**Table 1:** Genetic differentiation between populations as measured by median Weir–Cockerham weighted F_ST_ estimates

Population	Wamala^a^	Bukasa	Bugala	Sserinya	Nsadzi
Entebbe^a^	0.0342	0.0503	0.0457	0.0100	0.0444
Wamala^a^	–	0.0446	0.0412	0.0903	0.0389
Bukasa	–	–	0.0532	0.0800	0.0520
Bugala	–	–	–	0.0826	0.0480
Sserinya	–	–	–	–	0.0846

^a^Denotes mainland population

Generally, inter-island comparisons generated the greatest differences between populations with the strongest signals of genetic differentiation being observed in the comparisons with Sserinya (median F_ST_ > 0.08).

Geographical distance between populations is often the primary force driving genetic differentiation; therefore, isolation-by-distance as the model explaining the variation between the populations was tested by simple linear regression of median F_ST_/(1-median F_ST_) against a natural logarithm transformation of geographic distance [56]. The resulting GLM plot showed no evidence of a correlation between the two variables (*y *= 0.104975 − 0.012853*x*; R^2^ = 0.05; Mantel: p = 0.2) meaning that geographic distance alone could not explain the variation observed between the populations (Additional file 7).

### Effective population size

Estimations of N_e_ were obtained using the LDNe method [58] in NeEstimator v.2.01 [59] on the basis of superior performance compared to other single-sample estimators [59, 62, 63]. Generally, smaller estimates of N_e_ were observed for all populations (Table 2) compared to those recorded for other continental populations of *An. gambiae* i.e. 6689 (Kenya) [64], 13,200 (Equatorial Guinea) [65], 2 million (East Africa) [61].

**Table 2:** Estimates of effective population size (N_e_) using a minor allele frequency screen of 5%

Population	Bukasa	Bugala	Entebbe^a^	Nsadzi	Sserinya	Wamala^a^
No. individuals	13	13	16	16	5	16
% polymorphism	77	83	84	86	64	87
Harmonic mean sample size	8.0	9.4	13.8	14.6	4.7	15.0
Overall r^2^	0.17	0.14	0.09	0.09	0.37	0.08
Estimated N_e_	211.7	1098.3	213.5	124.2	∞	1920.3
95% CIs (parametric)	180.6–255.4	637.6–3913.7	195.8–234.6	118.4–130.7	∞	1124.2–6523.8
Coefficient of variation	0.005	0.001	0.005	0.008	∞	0.001

^a^Denotes mainland population

The largest estimates were seen in Bugala (1098.3) and Wamala (1920.3), which were substantially higher than the other populations. The infinity estimates recorded for the Sserinya populations were derived from negative points, which implied that variation was due to sampling error alone and not genetic drift (allelic frequency changes due to random sampling). This was unsurprising given the high levels of kinship observed in both populations as a result of the unrepresentative entomological sampling that necessitated the removal of eleven individuals from the dataset. The coefficient of variation is a measurement of genetic drift specific to the LDNe method of NeEstimator v2.01 [59] and is calculated as the inverse of N_e_. The lower estimates of 0.001 observed for Bugala and Wamala indicated that these populations would be more resistant to the effects of genetic drift compared to the higher coefficients of variation recorded for Bukasa (0.005), Entebbe (0.005) and Nsadzi (0.008), which implied vulnerability to allelic dropout or fixation.

## Discussion

### There are limited malaria vector species in the Ssese Islands

The majority of the dataset comprised of *An. gambiae* mosquitoes. Only 1 of 480 female anophelines was molecularly identified as *Anopheles arabiensis*, and this was from the mainland (Entebbe) population. Kayondo et al. [21] reported approximately 20% of the second year Bukasa collection (N = 47) as *An. arabiensis*, which was attributed to asynchronous entomological sampling. This observation highlights the importance of systematic population sampling to establish changes in (a) species composition, (b) vector abundance, and (c) seasonality, which are some of the factors that can influence the genetic structure, and effective size of a population.

### The 2La inversion confounds population genetic structure

Previous studies have demonstrated a strong association between the frequency of the 2La inversion and aridity, which shifts seasonally and geographically according to climate [66, 67], but there are no prior published data about its distribution in the Ssese Islands. Principal components analysis illustrated that the 2La inversion confounded population genetic structure (Fig. 2a, b), thus chromosome 2L SNPs were removed from the dataset to disclose the underlying population structure of the collinear genome (Fig. 2c).

### Moderate but significant genetic differentiation is observed in island populations

Genetic differentiation among the Ssese Islands was moderate in magnitude (median F_ST_ 0.0480–0.0846) but significantly greater than the very low differentiation between *An. gambiae* populations observed across opposite sides of continental Africa (mean F_ST_ 0.016) [15], and comparable in magnitude to populations separated by the KRVC (mean F_ST_ 0.104), which acts as a physical barrier to gene flow [17]. It would seem reasonable to suggest that water also acts as a physical barrier to gene flow in locations where it separates populations—just as the KRVC does further inland—since higher differentiation, and low amounts of gene flow were identified in oceanic island studies of *An. gambiae* in the Comoros (mean F_ST_ 0.199–0.250) [19], and of *An. arabiensis* in Madagascar, Reunion, and Mauritius (mean F_ST_ 0.169) [68]. Kayondo et al. [21] reported mean F_ST_ values of 0.014–0.105 in the same *An. gambiae* populations sampled here, which are of a lower magnitude than those in the oceanic island studies, but not unexpected given the smaller distances involved that allowed for frequent human-marine transportation routes or even wind-borne dispersal, which might have passively dispersed mosquitoes. This would suggest that water is not an absolute physical barrier to gene flow in this region as supported here by the evidence indicating limited migration between populations (Table 1; Fig. 2c) and greater genetic structure that is present in the islands. This was also observed in the PCA plot (Fig. 2c) where individuals clustered in concordance with their geographic origin. The two mainland sites, Entebbe and Wamala, also showed signs of population differentiation (median F_ST_ 0.0342) (Fig. 2c). Neither of these locations is separated by water, but the sampling point in Entebbe, Lunnyo, sits on the edge of a small harbour, which is separate from the rest of the town. There are no other obvious geographical barriers to gene flow between these sites so the explanation as to why the two mainland *An. gambiae* populations appear somewhat differentiated from each other must be due to other unknown factors of demographic, ecological or anthropogenic origin. A variety of molecular markers have been used to explore genetic differentiation in continental populations including microsatellites, mitochondrial DNA, allozymes, and SNPs [61, 69]. That all of these marker systems identify the same pattern of low genetic differentiation on the continent suggests that the heightened differentiation estimated in this study is not attributable to different marker systems, an assertion strengthened by the previous Ssese island studies of Kayondo et al. [21] and Lukindu et al. [70] using microsatellites and mtDNA, respectively.

### Island populations have small effective sizes

Higher levels of genetic differentiation could also be explained to some extent by the small estimates of N_e_ that were obtained through the LD method of NeEstimator v.2.01 [59]. N_e_ determines how random genetic drift affects the stability of allele frequencies in a population, which are more variable in smaller populations. Kayondo et al. [21] estimated that the island populations consisted of smaller demes in the hundreds (397–677) compared to the mainland populations that were in the thousands (8810–8935). This was anticipated since *An. gambiae* is usually found in close proximity to human habitation [66], and the collection sites in the islands are less intensely populated than those on the mainland. The estimates in this study are generally smaller but comparable to those of Kayondo et al. [21] (< 397), with the exception of Bugala, which has increased to 1098. Over the last few years, the human population size on Bugala has grown (from 34,800 in 2002 to 54,293 in 2016) as a result of economic development [71] and tourism. Human population growth on the island coupled with increased boat traffic to/from the mainland may have led to population growth of *An. gambiae*.

One of the key components of a population genetic analysis is the temporal stability of the population. A limitation of this study is that there is only one time point to estimate N_e_, which can fluctuate in accordance with climatic changes. Future research should; therefore, focus on monthly entomological sampling to account for variances in seasonal mosquito abundance [72, 73].

## Conclusions

This is the first genome-wide SNP-based study of *An. gambiae* population connectivity, and effective size in the Lake Victoria region. The island populations are comprised of a dominant malaria vector species (*An. gambiae*) with low to moderate genetic differentiation, and greater structure suggesting some limitation to migration between them. Smaller estimates of effective population size indicate that an introduced effector transgene should be susceptible to genetic drift but to ensure that it is driven to fixation instead of loss the construct would have to be paired with a robust gene drive mechanism.

Taking these findings into consideration, together with their favourable location and suitability for frequent monitoring, the Ssese Islands contain several candidate field locations, which merit further evaluation in regard of a potential GM mosquito pilot release.

## Additional files

**Additional file 1: Table S1.** GPS co-ordinates and distances between entomological sampling sites.

**Additional file 2: Table S2.** Bioinformatics pipeline detailing software programs and parameters used in the analysis of RADseq genomic data.

**Additional file 3: Table S3.** Kinship coefficient estimates and percentage missing data values per individual.

**Additional file 4: Table S4.** Chromosome 2L inversion molecular karyotype results.

**Additional file 5: Figure S1.** (a–d) PCA plots of *An. gambiae* chromosomes not shown in the main text.

**Additional file 6: Figure S2.** ADMIXTURE bar plot of chromosome 2L SNPs (*K* = 2) showing probable ancestry fractions of the six *An. gambiae* populations (N = 79) based on the 2La inversion.

**Additional file 7: Figure S3.** Generalized linear model plot of genetic differentiation against log geographic distance based on median F_ST._

## References

[1] WHO. World Malaria Report 2017. Geneva: World Health Organization; 2017. http://www.who.int/malaria/publications/world-malaria-report-2017/report/en/.

[2] The malERA Consultative Group on Vector Control (2011). A research agenda for malaria eradication: vector control. PLoS Med..

[3] The malERA Consultative Group on Drugs (2011). A research agenda for malaria eradication: drugs. PLoS Med..

[4] The malERA Consultative Group on Vaccines (2011). A research agenda for malaria eradication: vaccines. PLoS Med..

[5] Shaukat AM, Breman JG, McKenzie FE (2010). Using the entomological inoculation rate to assess the impact of vector control on malaria parasite transmission and elimination. Malar J..

[6] Holt RA, Subramanian GM, Halpern A, Sutton GG, Charlab R, Nusskern DR (2002). The genome sequence of the malaria mosquito *Anopheles gambiae*. Science.

[7] Lawniczak MK, Emrich S, Holloway AK, Regier AP, Olson M, White B (2010). Widespread divergence between incipient *Anopheles gambiae* species revealed by whole genome sequences. Science.

[8] Neafsey DE, Waterhouse RM, Collins FH, Emrich SJ, Fontaine MC, Gelbart W (2015). Highly evolvable malaria vectors: the genomes of 16 *Anopheles* mosquitoes. Science.

[9] Burt A (2014). Heritable strategies for controlling insect vectors of disease. Philos Trans R Soc B.

[10] Christophides GK (2005). Transgenic mosquitoes and malaria transmission. Cell Microbiol.

[11] Wang S, Jacobs-Lorena M (2013). Genetic approaches to interefere with malaria transmission by vector mosquitoes. Trends Biotechnol.

[12] Bian G, Joshi D, Dong Y, Lu P, Zhou G, Pan X (2013). *Wolbachia* invades *Anopheles stephensi* populations and induces refractoriness to *Plasmodium* infection. Science.

[13] Gantz VM, Jasinskiene N, Tatarenkova O, Fazekas A, Macias VM, Bier E (2015). Highly efficient Cas9-mediated gene drive for population modification of the malaria vector mosquito *Anopheles stephensi*. Proc Natl Acad Sci USA.

[14] Hammond A, Galizi R, Kyrou K, Simoni A, Siniscalchi C, Katsanos D (2016). A CRISPR–Cas9 gene drive system targeting female reproduction in the malaria mosquito vector *Anopheles gambiae*. Nat Biotechnol.

[15] Lehmann T, Hawley WA, Kamau L, Fontenille D, Simard F, Collins FH (1996). Genetic differentiation of *Anopheles gambiae* populations from East and West Africa: comparison of microsatellite and allozyme loci. Heredity.

[16] Besansky NJ, Lehmann T, Fahey GT, Fontenille D, Braack LE, Hawley WA (1997). Patterns of mitochondrial variation within and between African malaria vectors, *Anopheles gambiae* and *An. arabiensis*, suggest extensive gene flow. Genetics.

[17] Lehmann T, Hawley WA, Grebert H, Danga M, Atieli F, Collins FH (1999). The Rift Valley Complex as a barrier to gene flow for *Anopheles gambiae* in Kenya. J Hered.

[18] Lehmann T, Blackston CR, Besansky NJ, Escalante AA, Collins FH, Hawley WA (2000). The Rift Valley Complex as a barrier to gene flow for *Anopheles gambiae* in Kenya: the mtDNA perspective. J Hered.

[19] Marsden CD, Cornel A, Lee Y, Sanford MR, Norris LC, Goodell PB (2013). An analysis of two island groups as potential sites for trials of transgenic mosquitoes for malaria control. Evol Appl.

[20] Chen H, Minakawa N, Beier J, Yan G (2004). Population genetic structure of *Anopheles gambiae* mosquitoes on Lake Victoria islands, West Kenya. Malar J.

[21] Kayondo J, Mukwaya LG, Stump A, Michel AP, Coulibaly MB, Besansky NJ (2005). Genetic structure of *Anopheles gambiae* populations on islands in north-western Lake Victoria, Uganda. Malar J..

[22] VectorBase. 2017. http://biomart.vectorbase.org/biomart/martview/39809899a4a2f01f48761e0ae1fce499. Accessed 16 Nov 2017.

[23] Miller MR, Dunham JP, Amores A, Cresko WA, Johnson EA (2007). Rapid and cost-effective polymorphism identification and genotyping using restriction site associated DNA (RAD) markers. Genome Res.

[24] Baird NA, Etter PD, Atwood TS, Currey MC, Shiver AL, Lewis ZA (2008). Rapid SNP discovery and genetic mapping using sequenced RAD markers. PLoS ONE.

[25] Thomas AS (1941). The vegetation of the Sese Islands. Uganda: an illustration of edaphic factors in tropical ecology. J Ecol.

[26] Ssegawa P, Nkuutu DN (2006). Diversity of vascular plants on Ssese Islands in Lake Victoria, central Uganda. Afr J Ecol.

[27] National Environment Management Authority (NEMA). Kalangala District State of Environment Report, 2005. Kampala, Uganda: National Environment Management Authority (NEMA); 2005. p. 122. http://www.nemaug.org/district_reports/Kalangala_DSOER_2004.pdf.

[28] Uganda Bureau of Statistics. The National Population and Housing Census 2014-Main Report. Kampala, Uganda: Uganda Bureau of Statistics; 2016. p. 86. http://www.ubos.org/onlinefiles/uploads/ubos/NPHC/2014%20National%20Census%20Main%20Report.pdf.

[29] Uganda Ministry of Health. Uganda Malaria Quarterly Bulletin Issue 16: October–December 2016. Kampala: Uganda Ministry of Health; 2016 Dec p. 18. http://uphfp.org/?mdocs-file=2733.

[30] Uganda Bureau of Statistics, ICF. Uganda Demographic and Health Survey 2016: Key Indicators Report. Kampala, Uganda: Uganda Bureau of Statistics, and Rockville: ICF; 2018. p. 590. https://dhsprogram.com/pubs/pdf/FR333/FR333.pdf.

[31] Uganda Ministry of Health. National Malaria Control Program: newsletter-March 2018. Kampala: Uganda Ministry of Health; 2018. p. 20. http://health.go.ug/sites/default/files/LLIN%20News%20letter%20final.pdf.

[32] Gillies MT (1961). Studies on the dispersion and survival of *Anopheles gambiae* Giles in East Africa, by means of marking and release experiments. Bull Entomol Res.

[33] Gillies MT, de Meillon B (1968). The anophelinae of Africa south of the Sahara (Ethiopian zoogeographical region).

[34] Scott JA, Brogdon WG, Collins FH (1993). Identification of single specimens of the *Anopheles gambiae* complex by the polymerase chain reaction. Am J Trop Med Hyg.

[35] Chen H, Rangasamy M, Tan SY, Wang H, Siegfried BD (2010). Evaluation of five methods for total DNA extraction from Western Corn Rootworm beetles. PLoS ONE.

[36] Parchman TL, Gompert Z, Mudge J, Schilkey FD, Benkman CW, Buerkle CA (2012). Genome-wide association genetics of an adaptive trait in lodgepole pine. Mol Ecol.

[37] Max Planck Institute for Evolutionary Anthropology Bioinformatics Group. Tools for multiplex sequencing on the Illumina platform. https://bioinf.eva.mpg.de/multiplex/. Accessed 28 Feb 2014.

[38] White BJ, Santolamazza F, Kamau L, Pombi M, Grushko O, Mouline K (2007). Molecular karyotyping of the 2La inversion in *Anopheles gambiae*. Am J Trop Med Hyg.

[39] Babraham Institute. FastQC: a quality control tool for high-throughput sequence data. 2011. http://www.bioinformatics.babraham.ac.uk/projects/fastqc/. Accessed 15 Apr 2014.

[40] Bolger AM, Lohse M, Usadel B (2014). Trimmomatic: a flexible trimmer for Illumina sequence data. Bioinformatics.

[41] Notre Dame Bioinformatics Lab. Trimmer 2014. https://bitbucket.org/NDBL/hot-rad. Accessed 20 Feb 2015.

[42] VectorBase. Anopheles-gambiae-PEST_AgamP4_agp.gz. 2014. https://www.vectorbase.org/downloadinfo/anopheles-gambiae-pestchromosomesagamp4fagz. Accessed 30 Mar 2015.

[43] Li H, Durbin R (2009). Fast and accurate short read alignment with Burrows–Wheeler transform. Bioinformatics.

[44] Van der Auwera GA, Carneiro MO, Hartl C, Poplin R, del Angel G, Levy-Moonshine A (2013). From FastQ data to high-confidence variant calls: the Genome Analysis Toolkit best practices pipeline. Curr Protoc Bioinform..

[45] Fontaine MC, Pease JB, Steele A, Waterhouse RM, Neafsey DE, Sharakov IV (2015). Extensive introgression in a malaria vector species complex revealed by phylogenomics. Science.

[46] Danecek P, Auton A, Abecasis G, Albers CA, Banks E, DePristo MA (2011). The variant call format and VCFtools. Bioinformatics.

[47] Yang J, Benyamin B, McEvoy BP, Gordon S, Henders AK, Nyholt DR (2010). Common SNPs explain a large proportion of the heritability for human height. Nat Genet.

[48] Manichaikul A, Mychaleckyj JC, Rich SS, Daly K, Sale M, Chen W-M (2010). Robust relationship inference in genome-wide association studies. Bioinformatics.

[49] Chang CC, Chow CC, Tellier LC, Vattikuti S, Purcell SM, Lee JJ (2015). Second-generation PLINK: rising to the challenge of larger and richer datasets. GigaScience..

[50] R Core Team. R: a language and environment for statistical computing. Vienna: R Foundation for Statistical Computing; 2014. http://www.R-project.org/.

[51] Alexander DH, Novembre J, Lange K (2009). Fast model-based estimation of ancestry in unrelated individuals. Genome Res.

[52] Wright S (1978). Evolution and the genetics of populations: a treatise in four volumes: vol. 4: variability within and among natural populations.

[53] Weir BS, Cockerham CC (1984). F-Statistics for the analysis of population structure. Evolution.

[54] Arnold B, Corbett-Detig RB, Hartl D, Bomblies K (2013). RADseq underestimates diversity and introduces genealogical biases due to nonrandom haplotype sampling. Mol Ecol.

[55] Wright S (1943). Isolation by distance. Genetics.

[56] Rousset F (1997). Genetic differentiation and estimation of gene flow from F-Statistics under isolation by distance. Genetics.

[57] Mantel N (1967). The detection of disease clustering and a generalized regression approach. Cancer Res.

[58] Waples RS, Do C (2008). LDNE: a program for estimating effective population size from data on linkage disequilibrium. Mol Ecol.

[59] Do C, Waples RS, Peel D, Macbeth GM, Tillett BJ, Ovenden JR (2014). NeEstimator v2: re-implementation of software for the estimation of contemporary effective populations size (Ne) from genetic data. Mol Ecol Resour..

[60] Sharakhova MV, Hammond MP, Lobo NF, Krzywinski J, Unger MF, Hillenmeyer ME (2007). Update of the *Anopheles gambiae* PEST genome assembly. Genome Biol.

[61] O’Loughlin SM, Magesa S, Mbogo C, Mosha F, Midega J, Lomas S (2014). Genomic analyses of three malaria vectors reveals extensive shared polymorphism but contrasting population histories. Mol Biol Evol.

[62] Gilbert KJ, Whitlock MC (2015). Evaluating methods for estimating local effective population size with and without migration. Evolution.

[63] Wang J (2016). A comparison of single-sample estimators of effective population sizes from genetic marker data. Mol Ecol.

[64] Lehmann T, Hawley WA, Grebert H, Collins FH (1998). The effective population size of *Anopheles gambiae* in Kenya: implications for population structure. Mol Biol Evol.

[65] Athrey G, Hodges TK, Reddy MR, Overgaard HJ, Matias A, Ridl FC (2012). The effective population size of malaria mosquitoes: large impact of vector control. PLoS Genet.

[66] Coluzzi M, Sabatini A, Petrarca V, di Deco MA (1979). Chromosomal differentiation and adaptation to human environments in the *Anopheles gambiae* complex. Trans R Soc Trop Med Hyg.

[67] Touré YT, Dolo G, Petrarca V, Traoré SF, Bouaré M, Dao A (1998). Mark-release-recapture experiments with *Anopeheles gambiae* s.l. in Banambani Village, Mali, to determine population size and structure. Med Vet Entomol.

[68] Simard F, Fontenille D, Lehmann T, Girod R, Brutus L, Gopaul R (1999). High amounts of genetic differentiation between populations of the malaria vector *Anopheles arabiensis* from West Africa and eastern outer islands. Am J Trop Med Hyg.

[69] Anopheles gambiae 1000 Genomes Consortium (2017). Genetic diversity of the African malaria vector *Anopheles gambiae*. Nature..

[70] Lukindu M, Bergey CM, Wiltshire RM, Small ST, Bourke BP, Kayondo JK (2018). Spatio-temporal genetic structure of *Anopheles gambiae* in the north-western Lake Victoria basin, Uganda: implications for genetic control trials in malaria endemic regions. Parasit Vectors..

[71] Carmody P, Taylor D (2016). Globalization, land grabbing, and the present-day colonial state in Uganda: ecolonization and its impacts. J Environ Dev..

[72] Mukiama TK, Mwangi RW (1989). Seasonal population changes and malaria transmission potential of *Anopheles pharoensis* and the minor anophelines in Mwea irrigation scheme, Kenya. Acta Trop.

[73] Kabbale FG, Akol AM, Kaddu JB, Onapa AW (2013). Biting patterns and seasonality of *Anopheles gambiae* sensu lato and *Anopheles funestus* mosquitoes in Kamuli district, Uganda. Parasit Vectors..

