# Supplementary material for: Reduced-representation sequencing identifies small effective population sizes of Anopheles gambiae in the north-western Lake Victoria basin, Uganda
Source: Malar J. 2018 Aug 6;17:285. doi: 10.1186/s12936-018-2432-0 (PMC6080216; doi:10.1186/s12936-018-2432-0)
Supplement: Supplementary file 2 — Additional file 2: Table S2. Bioinformatics pipeline detailing software programs and parameters used in the analysis of RADseq genomic data. [file 12936_2018_2432_MOESM2_ESM.pdf]

Table S2. Bioinformatics pipeline detailing software programs and parameters used in the analysis of RADseq genomic data

Software	Version	Function	Parameters	Reference
(1) FastQC	0.10.1	Sequencing quality check		Babraham (2011)
(2) Trimmomatic	0.30	Remove Illumina sequencing adapters	ILLUMINACLIP:TruSeq3-PE.fa:2:30:10<br>LEADING:5<br>TRAILING:5<br>SLIDINGWINDOW:4:15<br>MINLEN:50	Bolger *et al.* (2014)
(3) Trimmer.py	Custom	RAD barcode, cut site and protector base removal; addition of unique IDs matching barcodes to individuals		Notre Dame Bioinformatics Lab (2014)
(4) Burrows-Wheeler Alignment (BWA)	0.6.2	Alignment of reads to reference genome	-t 12 -q 5 -l 32 -k 3 -n 9 -o 1	Li and Durbin (2009)
(5) sampToSam.pl	Custom	Addition of read groups (corresponding to unique sample IDs) to SAM files; change overall quality score for GATK compatibility		https://github.com/gjragland/perlScripts/blob/master/sampToSam.pl

Table S2. Bioinformatics pipeline detailing software programs and parameters used in the analysis of RADseq genomic data

(6) Picard Tools	1.119	Pre-processing of reads prior to variant calling	CleanSam<br>-VALIDATION_STRINGENCY=LENIENT<br>SortSam<br>-SO=coordinate<br>-VALIDATION_STRINGENCY=LENIENT<br>BuildBamIndex<br>-VALIDATION_STRINGENCY=LENIENT<br>CreateSequenceDictionary	http://broadinstitute.github.io/picard
(7) GenomeAnalysisToolKit (GATK)	3.3.0	Variant (SNP) calling	-T UnifiedGenotyper<br>--downsampling_type NONE<br>--downsample_to_coverage 1000<br>--genotype_likelihoods_model BOTH<br>--computeSLOD<br>-rf BadCigar<br>--fix_misencoded_quality_scores	Van de Auwera *et al.* (2013)
(8) GATK	3.3.0	Quality filtering of SNP calls; select SNPs that have passed filters and are biallelic only	-T VariantFiltration<br>--filterExpression "QD < 5.0 || FS > 60.0 || MQ < 40.0 || HaplotypeScore > 13.0 || MappingQualityRankSum < -12.5 || ReadPosRankSum < -8.0"<br>--missingValuesInExpressionsShouldEvaluateAsFailing<br>-T SelectVariants<br>--selectexpressions "vc.isNotFiltered() && vc.isSNP()"<br>--selectTypeToExclude INDEL<br>--restrictAllelesTo BIALLELIC	Van de Auwera *et al.* (2013)
(9) VCFtools	0.1.15	Pruning of dataset to include highest quality SNPs shared between populations	--mac 7 (at least 4 diploid individuals called at site)<br>--minGQ 30.0 (genotype quality of at least 30)<br>--minDP 30.0 (30 reads supporting called genotype)<br>--singletons<br>--max-missing 0.2 (proportion of missing data > 80%)	Danecek *et al.* (2011)
