# Supplementary material for: Reduced-representation sequencing identifies small effective population sizes of Anopheles gambiae in the north-western Lake Victoria basin, Uganda
Source: Malar J. 2018 Aug 6;17:285. doi: 10.1186/s12936-018-2432-0 (PMC6080216; doi:10.1186/s12936-018-2432-0)

Figure S1. (a-d) PCA plots of *An. gambiae* chromosome arms not shown in the main text

Each dot represents an individual mosquito (N=79) from one of the six sampling sites, which are color-coded according to the legend as follows: BK (Bukasa); BL (Bugala); EB (Entebbe); NZ (Nsadzi); SY (Sserinya), and WL (Wamala). The first principal component (PC1) and its percentage variance are represented on the *y*-axis, and, likewise, the second principal component (PC2) and its percentage variances are represented on the *x*-axis. *n* refers to the number of SNPs mapped to the chromosome arm using AgamP4 as a reference genome [42].

(a) Chromosome 2R after removal of the 2Rb inversion (n = 1,307)

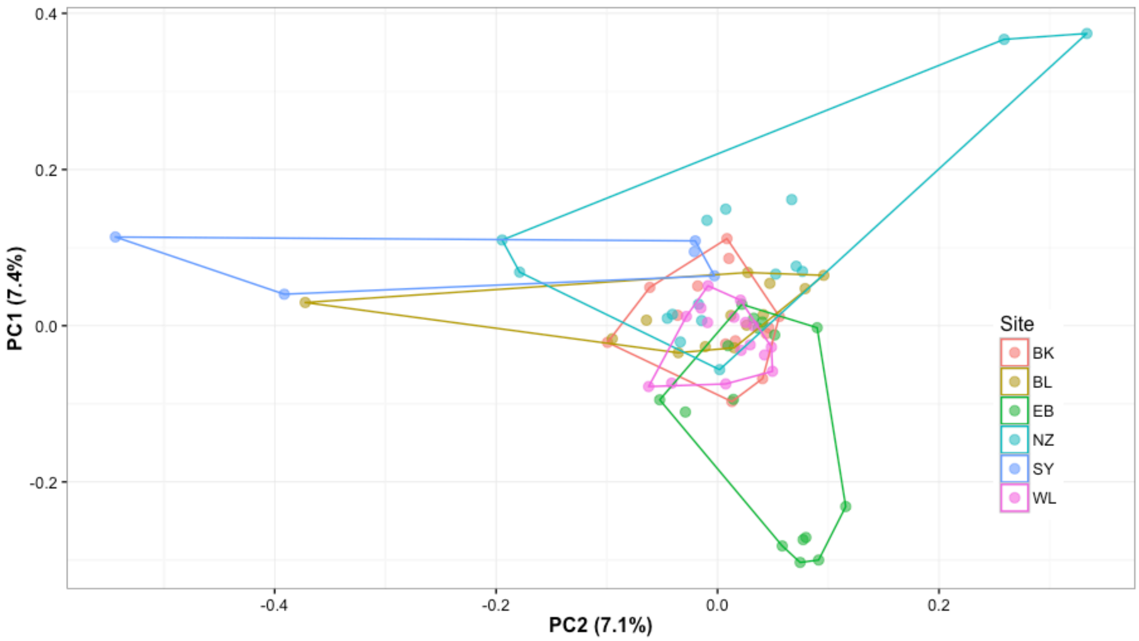

(b) Chromosome 3L (n = 936)

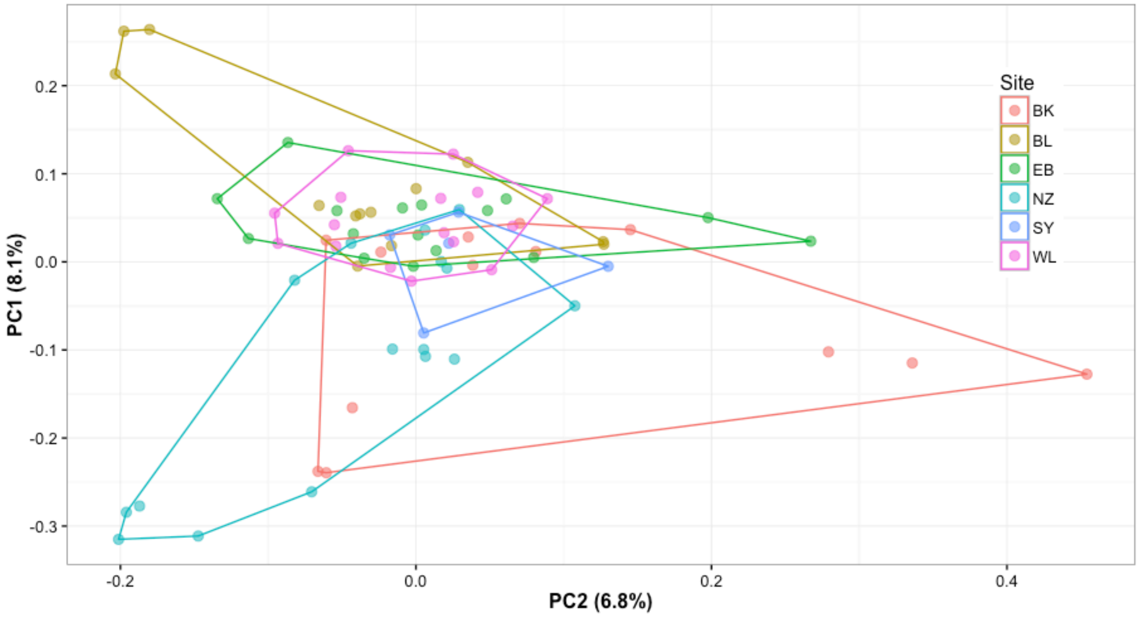

Figure S1. (a-d) PCA plots of *An. gambiae* chromosome arms not shown in the main text

(c) Chromosome 3R (n = 1,204)

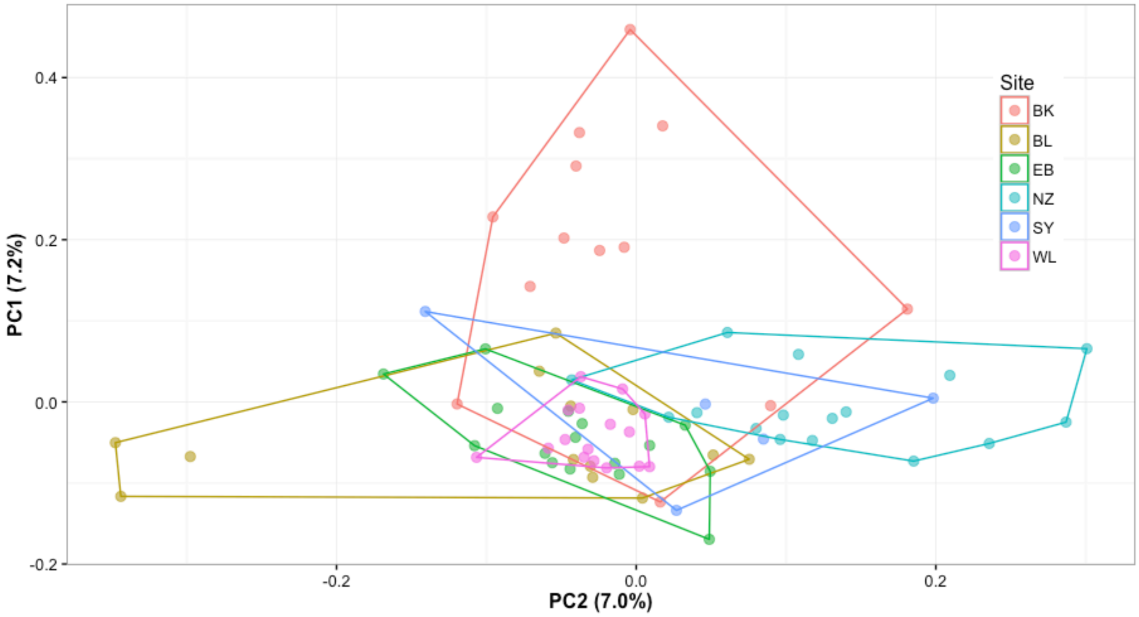

(d) Chromosome X (n = 347)

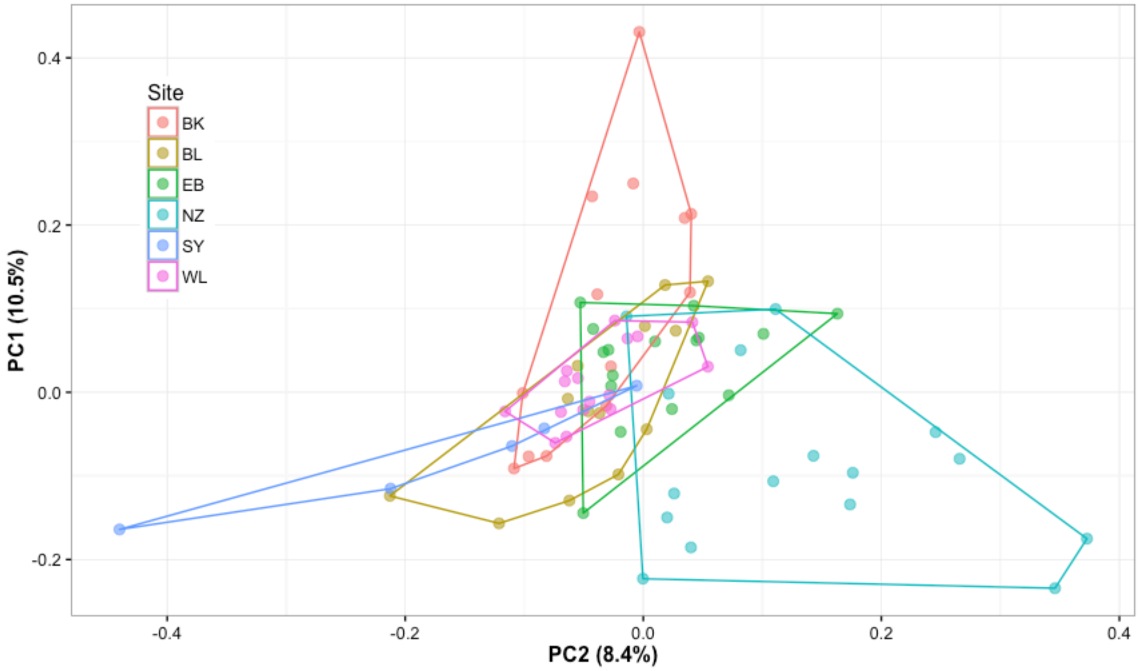

Supplement: Supplementary file 5 — Additional file 5: Figure S1. (a–d) PCA plots of An. gambiae chromosomes not shown in the main text. [file 12936_2018_2432_MOESM5_ESM.pdf]
