# Supplementary material for: Reduced-representation sequencing identifies small effective population sizes of Anopheles gambiae in the north-western Lake Victoria basin, Uganda
Source: Malar J. 2018 Aug 6;17:285. doi: 10.1186/s12936-018-2432-0 (PMC6080216; doi:10.1186/s12936-018-2432-0)

Figure S2. ADMIXTURE bar plot of chromosome 2L SNPs (*K*=2) showing probable ancestry fractions of the six *An. gambiae* populations (N=79) based on the 2La inversion. Vertical bars represent individual mosquitoes, which are stacked from left to right in groups of 16 (except: Bugala (N=13), Bukasa (N=13) and Sserinya (SY) (N=5)) according to their alphabetical sampling site. Populations are listed on the *x*-axis. Ancestry fractions (*K*=2) are assigned according to maximum likelihood probabilities. Each fraction represents a karyotype of different color: blue = inverted (2La/a), yellow = standard ($2L+^{a}/2L+^{a}$), and blue/yellow = heterozygote ($2La/+^{a}$).

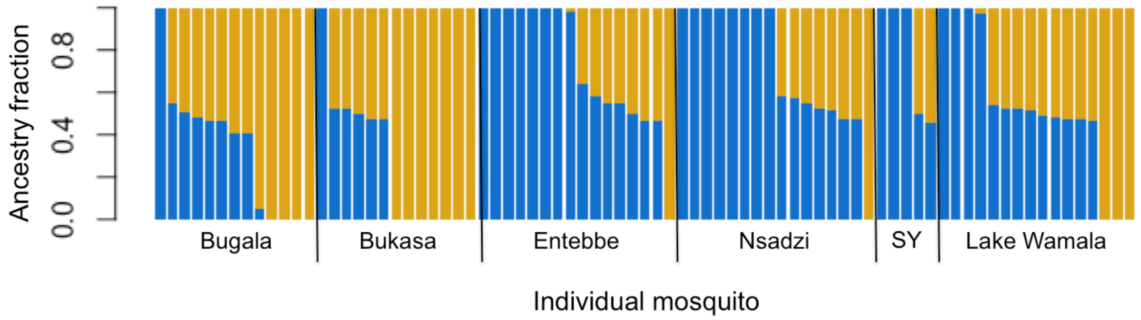

Supplement: Supplementary file 6 — Additional file 6: Figure S2. ADMIXTURE bar plot of chromosome 2L SNPs (K = 2) showing probable ancestry fractions of the six An. gambiae populations (N = 79) based on the 2La inversion. [file 12936_2018_2432_MOESM6_ESM.pdf]
