# Supplementary material for: Reduced-representation sequencing identifies small effective population sizes of Anopheles gambiae in the north-western Lake Victoria basin, Uganda
Source: Malar J. 2018 Aug 6;17:285. doi: 10.1186/s12936-018-2432-0 (PMC6080216; doi:10.1186/s12936-018-2432-0)

Figure S3. Generalized linear model plot of genetic differentiation against log geographic distance based on median F_{ST}.
The regression equation (y = 0.104975 - 0.012853x) describes the statistical relationship between the predictor ln(geographic distance) and response(Median F_{st}/(1-Median F_{st}) variables. The line of best fit is represented by the red dotted line.

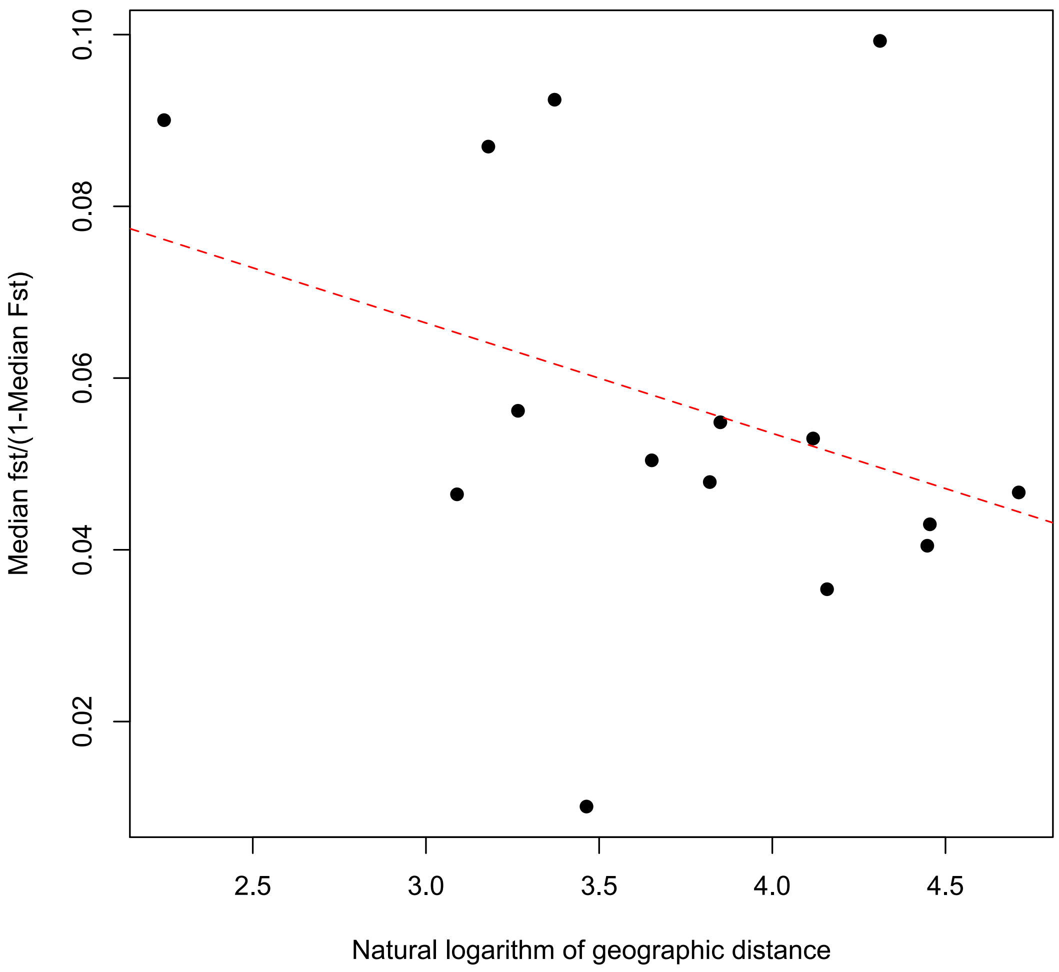

Supplement: Supplementary file 7 — Additional file 7: Figure S3. Generalized linear model plot of genetic differentiation against log geographic distance based on median FST. [file 12936_2018_2432_MOESM7_ESM.pdf]
